# The Mississippi Valley Medical Association

**Published:** 1889-12

**Authors:** 


The Mississippi Valley Medical Association at its 19th annual meeting held at Evansville, Ind., September 10, 11, 12, 1889, elected the following officers: President, Dr. Jos. M. Matthews, Louisville; 1st Vice-President, Dr. E. R. Early, Ridgway, Pa.; 2d Vice-President, Dr. Y. B. Harvey, Indianapolis, Ind.; Permanent Secretary, Dr. E. S. McKee, Cincinnati; Treasurer, Dr. C. F. McGahan, Chattanooga, Tenn.; Chairman Committee of Arrangements, Dr. J. N. Bloom, Louisville. Next meeting Louisville, September 9, 10, 11, 1890.

